# Supplementary material for: Splice-Junction-Based Mapping of Alternative Isoforms in the Human Proteome
Source: Cell Rep. Author manuscript; Available in PMC 2020 Jan 15. (PMC6961840; doi:10.1016/j.celrep.2019.11.026)

A

sp|Q15366|PCBP2_HUMAN|ENSG00000197111|SE1|9963|chr12|53462567|53464852|+0|r15|T1
YSTGSDSASFPHTTPSM[15.99]CLNPDLEGPPLELTK q value: 5.4871e−05 Tr_novel:TRUE RefSeq_Novel:TRUE
Search result spec prec mz: 1156.2035 Actual spec prec mz: 1156.2035
Fragments matched per AA: 0.969 Proportion of top 20 peaks matched: 0.35

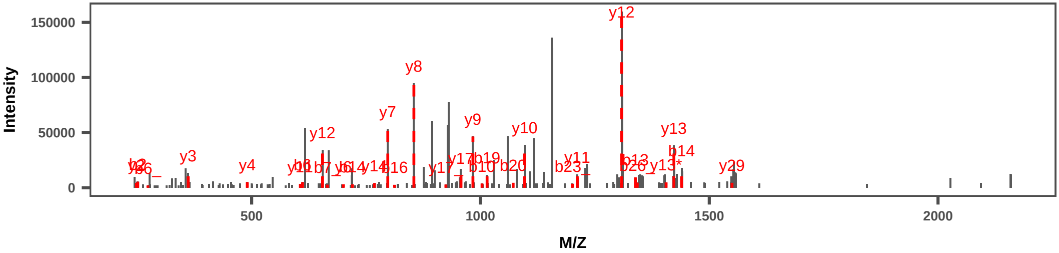

B

Scatterplot of predicted elution time
Fitting R2: 0.846
Novel peptide residual Z score: −0.964
Number of peptides: 2115

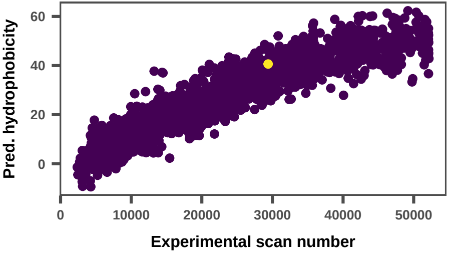

C

Distributions of residuals from best−fit line
of predicted RT vs Expt. scan number
Line: Z score of novel peptide
Z: −0.964

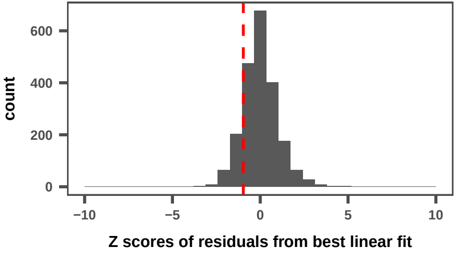

Supplement: 2 [file NIHMS1546469-supplement-2.zip › DF1/PXD010154/Spleen/Spleen_3_PCBP2_YSTGSDSASFPHTTPSMCLNPDLEGPPLELTK.pdf]
